# *KCNV2*-Associated Retinopathy: Detailed Retinal Phenotype and Structural Endpoints—*KCNV2* Study Group Report 2

**DOI:** 10.1016/j.ajo.2021.03.004

**Published:** 2021-10

**Authors:** Michalis Georgiou, Kaoru Fujinami, Ajoy Vincent, Fadi Nasser, Samer Khateb, Mauricio E. Vargas, Alberta A.H.J. Thiadens, Emanuel R. de Carvalho, Xuan-Thanh-An Nguyen, Thales Antônio Cabral De Guimarães, Anthony G. Robson, Omar A. Mahroo, Nikolas Pontikos, Gavin Arno, Yu Fujinami-Yokokawa, Shaun Michael Leo, Xiao Liu, Kazushige Tsunoda, Takaaki Hayashi, Belen Jimenez-Rolando, Maria Inmaculada Martin-Merida, Almudena Avila-Fernandez, Ester Carreño, Blanca Garcia-Sandoval, Carmen Ayuso, Dror Sharon, Susanne Kohl, Rachel M. Huckfeldt, Camiel J.F. Boon, Eyal Banin, Mark E. Pennesi, Bernd Wissinger, Andrew R. Webster, Elise Héon, Arif O. Khan, Eberhart Zrenner, Michel Michaelides

**Affiliations:** 1 Moorfields Eye Hospital, 162 City Rd, London EC1V 2PD, United Kingdom; 2 UCL Institute of Ophthalmology, University College London, 11-43 Bath St, London EC1V 9E, United Kingdom; 3 Laboratory of Visual Physiology, Division of Vision Research, National Institute of Sensory Organs, National Hospital Organization Tokyo Medical Center; 4 Department of Ophthalmology, Keio University School of Medicine, Tokyo, Japan; 5 Department of Ophthalmology and Vision Sciences, the Hospital for Sick Children, University of Toronto, Toronto, Ontario, Canada; 6 Institute for Ophthalmic Research, Centre for Ophthalmology, University of Tübingen, Tübingen, Germany; 7 Department of Ophthalmology, Hadassah Medical Center, Faculty of Medicine, the Hebrew University of Jerusalem, Jerusalem, Israel; 8 Department of Ophthalmology, Oregon Health & Science University, Casey Eye Institute, Portland, Oregon, USA; 9 Department of Ophthalmology, Erasmus Medical Center, Rotterdam; 10 Department of Ophthalmology, Amsterdam UMC, Academic Medical Center, Amsterdam; 11 Department of Ophthalmology, Leiden University Medical Center, Leiden, the Netherlands; 12 Department of Health Policy and Management, Keio University School of Medicine; 13 Department of Ophthalmology, Katsushika Medical Center, The Jikei University School of Medicine Tokyo, Japan; 14 Department of Ophthalmology, Instituto de Investigación Sanitaria-Fundación Jiménez Díaz University Hospital-Universidad Autónoma de Madrid (IIS-FJD, UAM), Madrid, Spain; 15 Department of Genetics, Instituto de Investigación Sanitaria-Fundación Jiménez Díaz University Hospital-Universidad Autónoma de Madrid (IIS-FJD, UAM); 16 Center for Biomedical Network Research on Rare Diseases (CIBERER), ISCIII, Madrid, Spain; 17 Department of Ophthalmology, Massachusetts Eye and Ear Infirmary, Harvard Medical School, Boston, Massachusetts, USA; 18 Eye Institute, Cleveland Clinic Abu Dhabi, Abu Dhabi, United Arab Emirates; 19 Department of Ophthalmology, Cleveland Clinic Lerner College of Medicine of Case Western University, Cleveland, Ohio, USA

## Abstract

**Purpose:**

To describe the detailed retinal phenotype of *KCNV2-*associated retinopathy.

**Study design:**

Multicenter international retrospective case series.

**Methods:**

Review of retinal imaging including fundus autofluorescence (FAF) and optical coherence tomography (OCT), including qualitative and quantitative analyses.

**Results:**

Three distinct macular FAF features were identified: (1) centrally increased signal (n = 35, 41.7%), (2) decreased autofluorescence (n = 27, 31.1%), and (3) ring of increased signal (n = 37, 44.0%). Five distinct FAF groups were identified based on combinations of those features, with 23.5% of patients changing the FAF group over a mean (range) follow-up of 5.9 years (1.9-13.1 years). Qualitative assessment was performed by grading OCT into 5 grades: (1) continuous ellipsoid zone (EZ) (20.5%); (2) EZ disruption (26.1%); (3) EZ absence, without optical gap and with preserved retinal pigment epithelium complex (21.6%); (4) loss of EZ and a hyporeflective zone at the foveola (6.8%); and (5) outer retina and retinal pigment epithelium complex loss (25.0%). Eighty-six patients had scans available from both eyes, with 83 (96.5%) having the same grade in both eyes, and 36.1% changed OCT grade over a mean follow-up of 5.5 years. The annual rate of outer nuclear layer thickness change was similar for right and left eyes.

**Conclusions:**

*KCNV2*-associated retinopathy is a slowly progressive disease with early retinal changes, which are predominantly symmetric between eyes. The identification of a single OCT or FAF measurement as an endpoint to determine progression that applies to all patients may be challenging, although outer nuclear layer thickness is a potential biomarker. Findings suggest a potential window for intervention until 40 years of age.

## Highlights

• *KCNV2*-associated retinopathy is a slowly progressive disease with early retinal changes, which are predominantly symmetric between eyes.
• Disease course can be unpredictable and may severely affect children and young adults.
• Findings suggest a potential window for intervention until 40 years of age, albeit with variability between patients due to macular atrophy.

*KCNV2*-associated retinopathy (cone dystrophy with supernormal rod responses; OMIM #610356) is an unusual autosomal recessive retinal disorder and a leading cause of cone-rod dystrophy.^1^, ^2^, ^3^ The *KCNV2*-retinopathy Study Group is a multicenter international retrospective investigation of clinically and molecularly confirmed patients with the disease. Report No. 1 of the study^4^ highlighted the early disease onset (<12 years of age), the severity of the clinical phenotype, the genetic background, and established a cohort of patients with a wide geographic distribution. Full-field electroretinograms (ERGs) are diagnostic and pathognomonic.^5^, ^6^, ^7^, ^8^, ^9^, ^10^, ^11^, ^12^ Report No. 1 showed a normal rate of age-associated ERG change, consistent with largely stable peripheral rod and cone system dysfunction across 6 decades.^4^ The purpose of the current study (Report No. 2) is to characterize the retinal architecture and the associated disease natural history.

A wide range of fundus autofluorescence (FAF) abnormalities including ring-like or bull's-eye changes, central atrophy, or increased foveal autofluorescence (AF) have been reported in *KCNV2*-retinopathy.^2^^,^^7^^,^^13^, ^14^, ^15^, ^16^ Optical coherence tomography (OCT) can show a variable degree of changes in the outer retina, ranging from ellipsoid zone (EZ) disruption to diffuse outer retinal atrophy with preservation of the inner nuclear layers, usually limited to the macula.^12^^,^^15^^,^^17^, ^18^, ^19^, ^20^ In a cross-sectional study of 18 patients, Sergouniotis and associates^17^ identified 4 foveal OCT phenotypes: (1) discontinuous EZ (n = 6); (2) loss of EZ and an optical gap at the foveola (n = 2); (3) EZ disruption and profound foveal depth reduction, without optical gap and with preserved retinal pigment epithelium (RPE) complex (n = 2); and (4) outer retina and RPE complex abnormalities (n = 2). The literature describing the retinal phenotype of the disease is limited to cross-sectional studies, or small longitudinal cohorts and case reports.^2^ *KCNV2*-retinopathy is a potential target for trial of novel therapeutic interventions, such as gene augmentation therapy,^21^ to restore outer retinal function before the advent of macular atrophy.

The *KCNV2*-retinopathy Study Group is the first multicenter international collaborative retrospective study in a large cohort (n = 117) of adults and children with *KCNV2*-retinopathy. Herein, we present Report No. 2, which provides a detailed analysis of retinal imaging both cross-sectionally and longitudinally. Retinal phenotyping is a crucial step toward the design of a prospective natural history study, and of paramount importance for the feasibility and the success of any upcoming therapeutic clinical trial in *KCNV2*-retinopathy, as well as identifying clinically meaningful and reliable structural endpoints.

## METHODS

The study protocol adhered to the tenets of the Declaration of Helsinki and received approval from all local ethics committees of the participating institutions. Informed consent was obtained from all adult patients, whereas informed consent and assent were obtained from parents and children, respectively, as indicated.

### Patient Identification

Inclusion criteria for the current study were the molecular and phenotypic confirmation of likely biallelic *KCNV2*-retinopathy, as described in Report No. 1.

### Retinal Imaging

FAF and OCT imaging was performed using different imaging systems across sites (FAF: Spectralis Heidelberg Engineering Ltd, Heidelberg, Germany; Optos plc, Dunfermline, United Kingdom; OCT: Spectralis Heidelberg Engineering Ltd; Cirrus HD OCT, version 6.5, Carl Zeiss Meditec, Jena, Germany; DRI OCT, Topcon, Tokyo, Japan), and was used to assess cross-sectional and longitudinal structural changes.

Qualitative FAF and OCT analysis was performed using all available data from all participating institutions, at baseline imaging and last follow-up. Quantitative analysis was performed in all patients seen in a single referral center (Moorfields Eye Hospital, London, United Kingdom), by a single observer, and imaged using a single imaging system (Spectralis; Heidelberg Engineering Ltd), in order to avoid variability among systems and observers.

### FAF Analysis

All FAF images were examined for the pattern of AF and the presence of a hyperautofluorescent ring. In patients displaying a hyperautofluorescent ring and/or a well-demarcated region of decreased AF (DAF), quantitative analysis was performed. The ring area of increased signal was calculated by subtracting from the area delineated by the outer border of the hyperautofluorescent ring, the aforementioned central area of DAF. The Heidelberg Spectralis Region Finder tool was used for semiautomated quantitative analysis on 30 degree × 30 degree images, as previously described (Supplemental Material: Methods).^22^, ^23^, ^24^, ^25^, ^26^, ^27^, ^28^ The baseline measurement of DAF and the rate of progression (mm^2^/y) were evaluated for disease symmetry between eyes. The annual rate of progression was calculated.

### OCT Analysis

**Figure 1:**
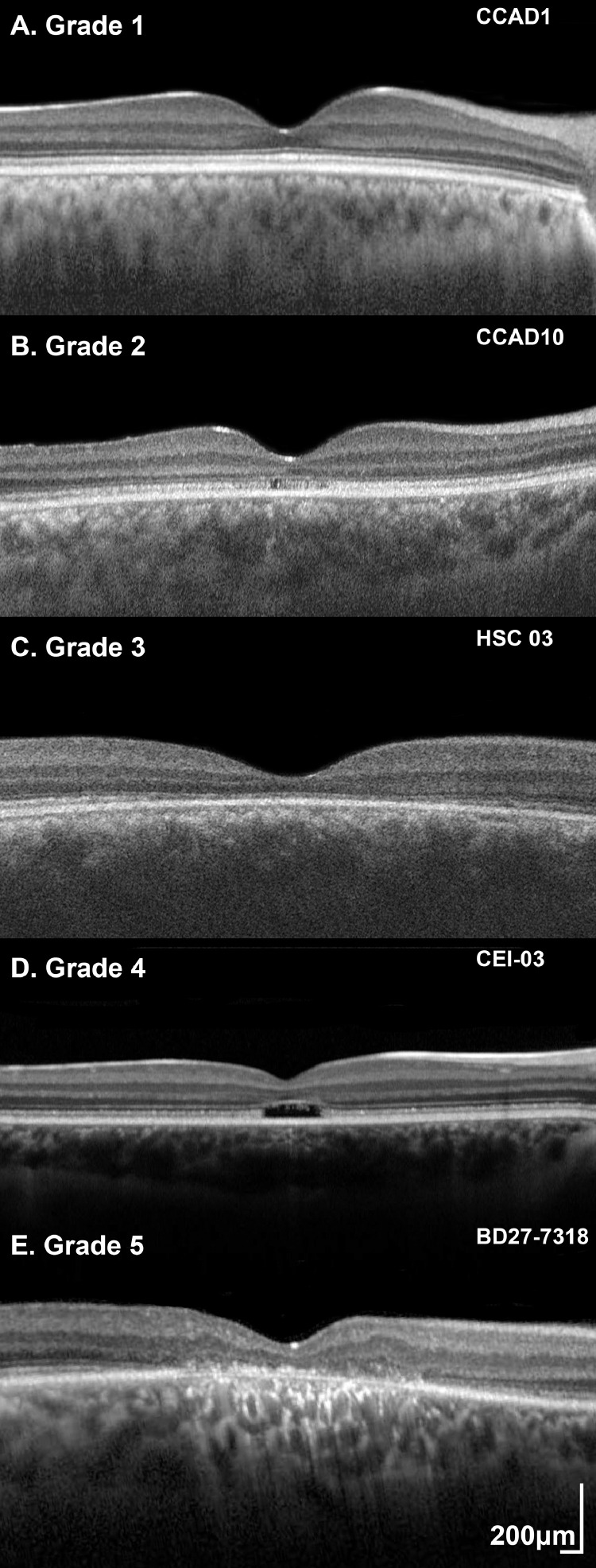
Optical coherence tomography (OCT) grading system. Qualitative assessment of foveal structure was performed by grading transfoveal horizontal OCT images into 1 of 5 foveal OCT grades: (A) Grade 1: continuous ellipsoid zone (EZ), (B) Grade 2: EZ disruption, (C) Grade 3: EZ absence, without optical gap and with preserved retinal pigment epithelium (RPE) complex, (D) Grade 4: loss of EZ and a hyporeflective zone (optical gap) at the foveola, and (E) Grade 5: outer retina and RPE complex loss.

Qualitative assessment of foveal structure was performed by grading spectral domain-OCT images into 1 of 5 foveal OCT grades: (1) continuous EZ; (2) EZ disruption; (3) EZ absence, without optical gap and with preserved RPE complex; (4) loss of EZ and a hyporeflective zone (optical gap) at the foveola; and (5) outer retina and RPE complex loss (Figure 1). For each patient, both right and left eyes were graded at baseline and follow-up. The presence/absence of foveal hypoplasia was also noted, defined as the persistence of 1 or more inner retinal layers (outer plexiform layer, inner nuclear layer, inner plexiform layer, or ganglion cell layer) through the fovea, as previously applied in the cone dysfunction syndromes.^29^, ^30^, ^31^

**Figure 2:**
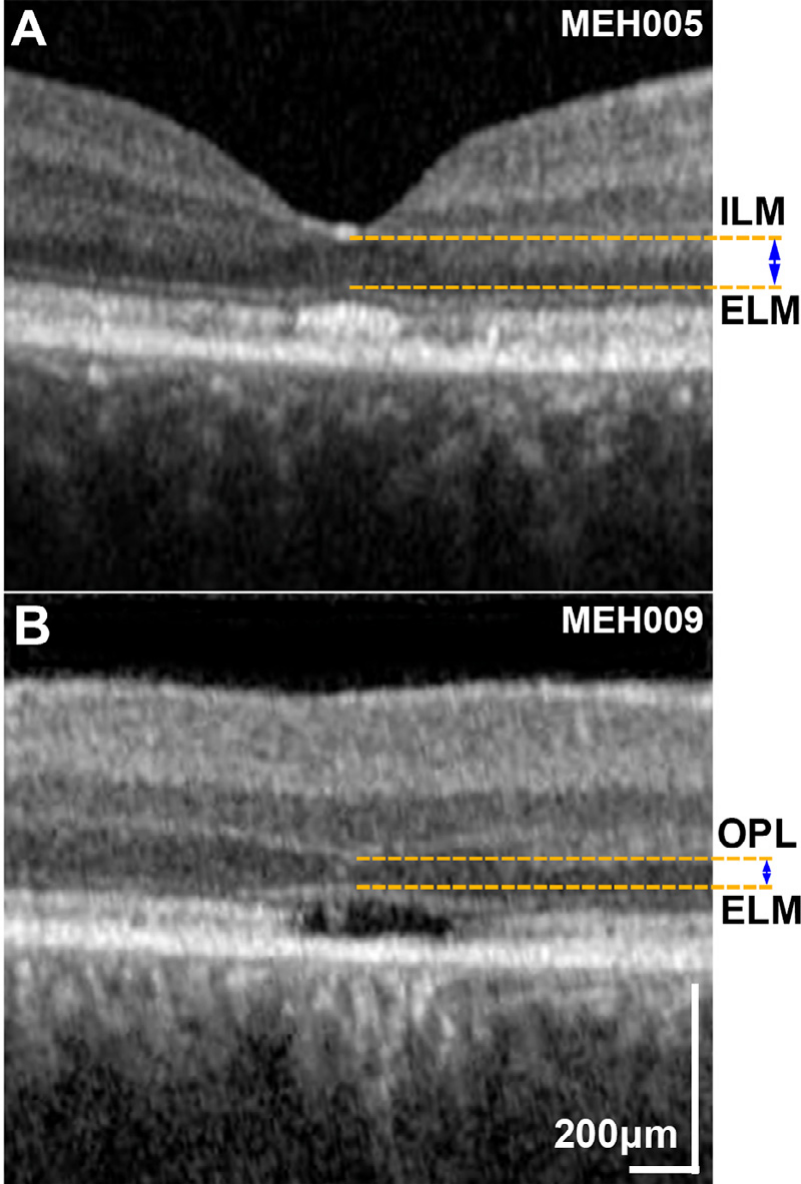
Outer nuclear layer (ONL) thickness measurement. Selected foveal optical coherence tomography horizontal scans of patients with *KCNV2*-retinopathy: (A) without foveal hypoplasia and (B) with foveal hypoplasia. (A) The ONL thickness was calculated in patients without foveal hypoplasia, as the distance between the internal limiting membrane (ILM) and the external limiting membrane (ELM). (B) In patients with foveal hypoplasia, ONL thickness was calculated as the distance between the outer plexiform layer (OPL) and ELM.

Quantitative analysis was performed using digital calipers (Heidelberg Eye Explorer; Heidelberg Engineering), and a 1-μm:1-μm display with maximum magnification, on the transfoveal horizontal line scan, with the foveal reflex used as an anatomical landmark. The extent of the central EZ disruption was measured after marking the nasal and temporal boundaries of the EZ lesion. The outer nuclear layer (ONL) thickness was calculated in patients without foveal hypoplasia as the distance between the internal limiting membrane and the external limiting membrane, also using the digital calipers. In patients with foveal hypoplasia, ONL thickness was calculated as the distance between the outer plexiform layer and the external limiting membrane. Figure 2 shows examples of patients with and without foveal hypoplasia. Follow-up mode was used, so that the same scanning location was imaged at follow-up and baseline. In addition, the methods described by Tee and associates^32^ were employed to ensure serial analysis of the same patient-specific retinal location (Supplemental Material: Methods).

### Statistical Methods

Statistical analysis was performed with IBM SPSS Statistics for Windows (Version 22.0; IBM Corp., Armonk, New York, USA). The Shapiro-Wilk test of normality was used for all variables, and parametric or nonparametric tests were used accordingly.

## RESULTS

The demographics and genetics of the cohort were described in detail in Report No. 1. The number of patients with available data, baseline age, and the follow-up time are indicated below on each individual assessment.

### Qualitative FAF Analysis

**Figure 3:**
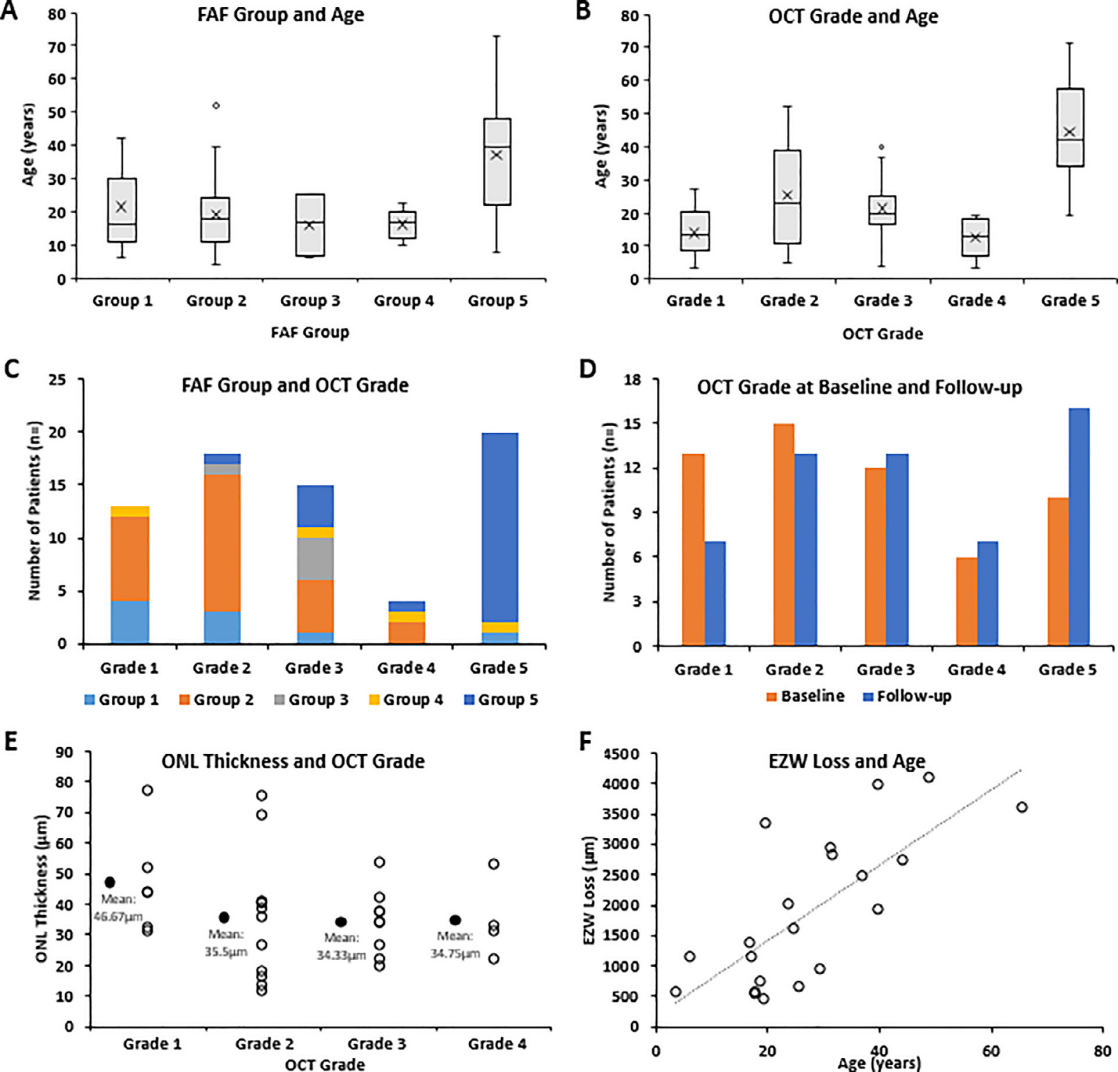
Fundus autofluorescence (FAF) and optical coherence tomography (OCT) data analysis and graphs. (A) Boxplots for the age of each identified FAF group. The X mark represents the mean for each group. The mean age for Group 5 was the highest, but there was an age overlap between groups. (B) Boxplots for the age of each identified OCT grade. The X mark represents the mean for each group. The mean age for Grade 5 was the highest, but there was an age overlap between grades similar to FAF groups. (C) The graph presents the number of patients for each OCT grade, and what the FAF group of those patients was. Lower OCT grades (1 and 2) had mostly FAF Groups 1 and 2, and OCT Grade 5 had in the vast majority FAF Group 5. (D) OCT grading at baseline (orange) and follow-up (blue) for 56 patients, with a mean follow-up time (±standard deviation, range) of 4.38 years (±2.82, 0.3-11 years). (E) Stacked scatter plot on the outer nuclear layer (ONL) thickness of patients with OCT Grades 1-4. The black dots represent the mean of each grade. All grades had severely reduced ONL thickness. (F) The mean ellipsoid zone width (EZW) loss for right and left eyes is plotted against age. There was a strong positive statistically significant correlation between the EZW loss and age (*r* = 0.72, *P* < .000, Spearman).

**Figure 4:**
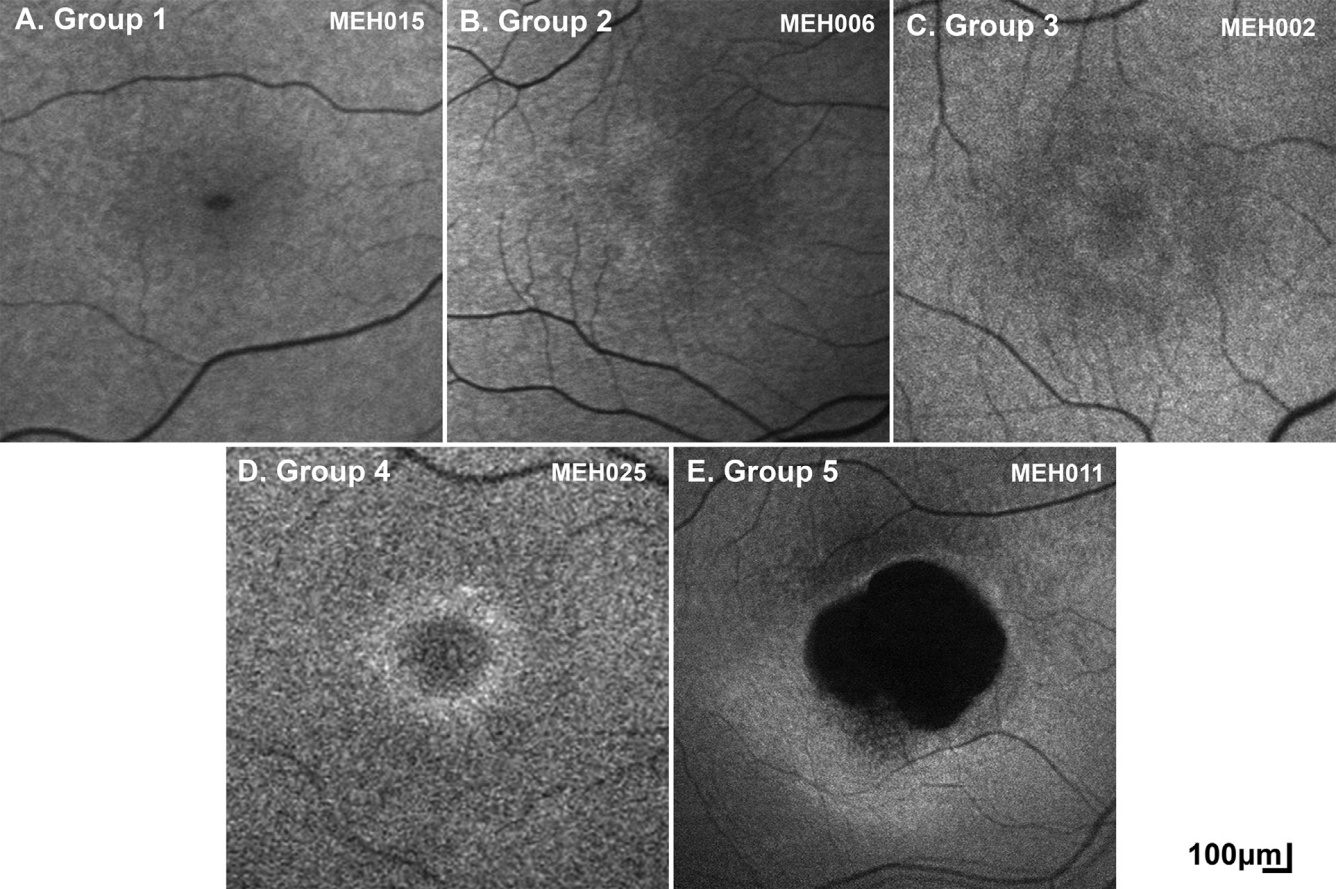
Fundus autofluorescence (FAF) in *KCNV2*-retinopathy. Based on the presence of (1) increased autofluorescence (AF), (2) decreased autofluorescence (DAF), and/or (3) hyperautofluorescent perimacular ring, 5 groups/patterns of FAF were identified. (A) Group 1: negative for all 3 features, (B) Group 2: increased central signal, (C) Group 3: perimacular ring and centrally increased AF, (D) Group 4: perimacular ring, and (E) Group 5: DAF and ring.

Eighty-four patients had a baseline FAF assessment. Three distinct macular features were identified: (1) increased signal (n = 35, 41.7%), (2) DAF (n = 27, 31.1%), and (3) ring of increased signal (n = 37, 44.0%). The aforementioned characteristics form 5 distinct groups of FAF patterns: (1) Group 1: negative for all 3 features, (2) Group 2: increased central signal, (3) Group 3: perimacular ring and centrally increased AF, (4) Group 4: perimacular ring without centrally increased AF, (5) Group 5: DAF and perimacular ring. The FAF groups are summarized in Table 1 and in the boxplots (Figure 3, A). Eighty-two patients had bilateral data, with both eyes showing the same FAF pattern. Examples of the 5 FAF groups are presented in Figure 4.

**Table 1:** Qualitative Fundus Autofluorescence (FAF) and Optical Coherence Tomography (OCT) Analysis

FAF^a^						
Parameter	Group 1	Group 2	Group 3	Group 4	Group 5	Cohort
Number of patients, n (%)	16 (19.0)	31 (36.9)	4 (4.8)	6 (7.1)	27 (32.1)	84
Mean age (y)	21.6	19.4	16.2	16.4	37.0	25.1
Median age (y)	16.5	18.0	16.6	16.9	39.8	21.0
Age range (y)	6-42	4-52	6-25	10-23	8-73	4-73
Increased macular autofluorescence, n (%)	No	Yes	Yes	No	No	35 (41.7)
Decreased macular autofluorescence, n (%)	No	No	No	No	Yes	27 (32.1)
Perimacular ring, n (%)	No	No	Yes	Yes	Yes	37 (44.0)

OCT^a^						
Parameter	Grade 1	Grade 2	Grade 3	Grade 4	Grade 5	Cohort

Number of patients, n (%)	18 (20.5)	23 (26.1)	19 (21.6)	6 (6.8)	22 (25.0)	88
Mean age (y)	14	25.5	21.4	12.4	43.3	25.8
Median age (y)	13.5	23.0	19.8	13.0	40.0	21
Age range (y)	4-27	5-52	4-41	4-19	19-71	4-71

a Results presented are for the right eyes; 82/82 patients had similar FAF findings and 83/86 patients had similar OCT findings, in the fellow eye.

Follow-up data were available for 47 patients, with a mean follow-up (±standard deviation [SD], range) of 5.4 years (±3.6, 0.3-13.8 years). Thirteen patients were graded as Group 5 at baseline (mean age: 35.8 years). Of the remaining 34 patients (Groups 1-4), 26 patients (76.5%) preserved the same FAF group and 8 patients (23.5%) changed the FAF group over follow-up. Five patients changed from Group 1 to Group 2 (n = 2), Group 3 (n = 1), Group 4 (n = 1), and Group 5 (n = 1), and 2 patients changed from Group 2 to Group 4 (n = 1) and Group 5 (n = 1). One patient progressed from Group 4 to Group 5. The mean (range) follow-up in the patients with and without change of the FAF group was 4.9 years (0.3-12.3 years) and 5.9 years (1.9-13.1 years), respectively. The mean baseline age for the patients with and without progression was 17.0 and 18.7 years, respectively.

### Quantitative FAF Analysis

Seven patients had quantifiable DAF and/or a ring of increased signal areas, with a mean (range) age of 39.3 years (19.3-59.8 years). Quantitative FAF measurements are summarized in Table 2. The measurements of baseline DAF area, baseline ring area, and their annual rate of progression were not significantly different between eyes (*P* = .56, *P* = .29, *P* = .70, *P* = .12; *t* = −0.62, *t* = −1.16, *t* = 0.41, and *t* = 1.80 respectively, *df* = 6, paired *t* test).

**Table 2:** Quantitative Fundus Autofluorescence and Optical Coherence Tomography Analysis^a^

Fundus Autofluorescence (n = 7)	
Parameter	Mean, ±SD, Range
Perimacular ring area at baseline	
Right eye	2.18, ±1.22, 0.24-4.65 mm^2^
Left eye	2.47, ±1.54, 0.28-5.52 mm^2^
Ring area annual rate of change	
Right eye	0.23, ±0.14, 0.05-0.46 mm^2^/y
Left eye	0.14, ±0.13, −0.04 to 0.39 mm^2^/y
DAF area at baseline	
Right eye	2.17, ±1.51, 0.08-4.55 mm^2^
Left eye	2.38, ±1.87, 0.07-5.94 mm^2^
DAF annual rate of change	
Right eye	0.14, ±0.11, 0.00-0.31 mm^2^/y
Left eye	0.12, ±0.12, −0.04 to 0.31 mm^2^/y
Optical Coherence Tomography	
ONL thickness at baseline (n = 31)	
Right eye	38.26, ±14.64, 14-70 μm
Left eye	37.23, ±16.14, 12-77 μm
Grade 1 (n = 6)^b^	46.67, ±15.40, 31-77 μm
Grade 2 (n = 12)^b^	35.50, ±19.54, 12-75 μm
Grade 3 (n = 9)^b^	35.33, ±10.24, 19-59 μm
Grade 4 (n = 4)^b^	35.25, ±12.19, 20-54 μm
ONL thickness annual rate of change (n = 25)	
Right eye	−2.22, ±4.12, −13.62 to 5.69 μm/y
Left eye	−1.45, ±4.63, −14.25 to 9.16 μm/y
EZW loss at baseline (n = 21)	
Right eye	1890, ±1237, 437-4318 μm
Left eye	1873, ±1168, 439-3995 μm
Grade 3 (n = 9)^b^	1301, ±763, 570-3090 μm
Grade 4 (n = 4)^b^	748, ±384, 439-1403 μm
Grade 5 (n = 8)^b^	3080, ±626, 1924-3995 μm
EZW loss annual rate of change (n =17)	
Right eye	−110, ±126, −511 to 3 μm/y
Left eye	−94, ±116, −476 to 7 μm/y

DAF = decreased autofluorescence, EZW = ellipsoid zone width, ONL = outer nuclear layer.

a There is no statistically significant difference for any of the examined measurements (*P* > .05), between right and left eyes.

b Values were calculated for left eyes.

### Qualitative OCT Analysis

Eighty-eight patients had a baseline OCT assessment (mean age, range; 25.8 years, 4-71 years). Qualitative assessment of foveal structure was performed by grading SD-OCT images into 1 of 5 foveal OCT grades (Figure 1), as presented in Table 1 and Figure 3, B. Eighty-six patients had scans available from both eyes, with 83 (96.5%) having the same grade in both eyes. An example of disease asymmetry is presented in Supplemental Figure 1. Foveal hypoplasia was identified in 29 patients (33.0%) and was present in both eyes. For 70 patients, OCT and FAF imaging was available at the same visit. The graph in Figure 3, C, presents how FAF groups relate to OCT grades. Lower OCT grades (1 and 2) had mostly FAF groups 1 and 2, and OCT Grade 5 had in the vast majority FAF group 5.

Follow-up data were available for 56 patients, with a mean follow-up time (±SD, range) of 4.38 years (±2.82, 0.3-11 years). Ten patients were Grade 5 at baseline (mean age: 47.4 years). Of the remaining 46 patients (Grades 1-4), 28 patients (60.1%) preserved the same OCT grade and 18 patients (36.1%) changed OCT grade. Six patients changed from Grade 1 to Grade 2 (n = 5) and Grade 4 (n = 1), and 7 patients changed from Grade 2 to Grade 3 (n = 6) and Grade 5 (n = 1). Five patients progressed from Grade 3 to Grade 5. The mean (range) follow-up in the patients with and without change of OCT grade was 5.46 years (1-10 years) and 3.51 years (0.3-9.2 years), respectively. The mean baseline age for the patients with and without progression was 19.5 and 18.4 years, respectively. One patient with asymmetry at baseline preserved the asymmetry over time (Supplemental Figure 1), and another patient developed different grades between eyes during follow-up.

### Quantitative OCT Analysis

The ONL thickness was quantified in 31 patients (mean age, range: 20.7, 3.6-52.1 years). All patients had OCT Grades 1-4. Patients with OCT Grade 5 by definition had the ONL thickness of 0 μm because the outer retina is lost. Figure 3, E, presents the ONL thickness for each group and patient. The values were similar for right and left eyes (P= .27, *t* = 1.13, *df* = 30, paired *t* test). There was no statistically significant correlation between baseline age and mean ONL thickness for right and left eyes (*P* = .40, *r* = –0.16, Pearson).

Twenty-five patients (mean age, range: 22.4, 3.6-52.1 years) had longitudinal ONL thickness measurements. The mean (±SD, range) follow-up time was 3.8 years (±2.51, 0.4-9.2 years). The annual rate of ONL thickness change was also similar for right and left eyes (*P* = .12, *Z* = −1.55, Wilcoxon matched-pairs test): −2.22 and −1.45 μm/y, respectively. Table 2 summarizes ONL thickness baseline measurements for right and left eyes, and individual grades, and the annual rate of change.

In 21 patients (mean age, range: 27.5, 3.6-65.6 years), the EZ width (EZW) loss was quantified. All patients had OCT Grades 3-5. By definition, patients with Grades 1 and 2 lack EZW loss. The mean EZW loss for right and left eyes and each individual grade are summarized in Table 2. The EZW loss was not significantly different for right and left eyes (P= .60, *Z* = −0.521, Wilcoxon matched-pairs test). There was a strong positive statistically significant correlation between the EZW loss and age (*r* = 0.72, *P* < .000, Spearman, Figure 3, F).

Longitudinal EZW loss was evaluated for 17 patients (mean age, range: 28.8, 3.6-65.6 years) over a follow-up period of 1.3 to 9 years (mean: 4.1 years). The rate of EZW loss is recorded in Table 2, and it was similar between eyes (P = .10, *Z* = −1.63, Wilcoxon matched-pairs test). There was no statistically significant correlation between the mean rate of change for right and left eyes, and the baseline age (*r* = 0.16, *P* = .54, Spearman) or the mean baseline EZW loss (*r* = 0.08, *P* = .75, Spearman).

## DISCUSSION

We investigated the retinal phenotype and potential structural endpoints, both cross-sectionally and longitudinally, in a large cohort of patients with *KCNV2*-retinopathy, over a wide range of ages. The retinal phenotype is broadly in keeping with previous reports, and we were able to comprehensively quantify and qualify OCT and FAF features. We identified a wide therapeutic window for planned and anticipated interventional trials, variable and slowly progressive structural changes, which may be a challenge for the identification of primary anatomical outcomes.

### Disease Natural History

In the largest FAF study of the disease to date (cross-sectional, n = 24), the identification of small rings of increased signal was suggested as a nonspecific early manifestation of macular dysfunction.^7^ Herein, empowered by the far larger number of patients and the available imaging in patients younger than the age of 10, we were able to identify patients with no obvious retinal abnormalities on FAF and OCT imaging. We suggest a model for the FAF phenotype, starting with a “normal” appearance (Group 1, Figure 4, A), progressing to a centrally not well-defined increased signal (Group 2, Figure 4, B), then a perimacular ring of increased signal forms (Group 3, Figure 4, C), and with time, the central increased signal shortly decreases similar to normal and is surrounded by a hyperfluorescent ring (Group 4, Figure 4, D), and finally the foveal center loses its signal (atrophy) and both the atrophic area and the ring increase centrifugally over time (Group 5, Figure 4, E). Similarly, OCT changes can start with continuous EZ (Grade 1, Figure 1, A), followed by EZ disruption (Grade 2, Figure 1, B), then EZ absence (Grade 3, Figure 1, C) or loss of EZ and a hyporeflective zone (Grade 4, Figure 1, D), and finally outer retina and RPE complex loss (Grade 4, Figure 1, E). The continuous appearance of the EZ does not translate to the normal outer retina structure because of the severely decreased ONL thickness (Figure 3, E), in agreement with a previous report by Sergouniotis and associates.^17^ The low proportion of patients with FAF Groups 3 and 4, and OCT Grades 3 and 4 (Table 1) in our study, can support the speculation that those stages are short transitional state of the disease natural history, in contrast to *CNGB3*-, *CNGA3*-, and *PDE6C*-achromatopsia where Grade 4 is more common.^29^^,^^30^^,^^33^ *KCNV2*-retinopathy is a slowly progressive disease with early retinal changes.

### Disease Symmetry

All the parameters examined in the study, both qualitative and quantitative, were evaluated for disease symmetry. The quantitative FAF (DAF area, baseline ring area, and annual rate of progression) and OCT (EZW loss, rate of EZW loss, and ONL thickness) parameters were not significantly different between eyes, as well as the qualitative assessment for the FAF group (100%) and OCT grade (96.5%). The identified disease symmetry suggests similar therapeutic potential for both eyes, and it is of value for randomization in clinical trials, with the fellow eye serving as control for the treated eye. However, rare instances of asymmetry (Supplemental Figure 1**)** should be considered when stratifying patients and evaluating the outcomes of future clinical trials and were only observed with OCT imaging. Disease symmetry can be a screening inclusion criterion for a trial. In this respect, *KCNV2*-retinopathy is similar to other inherited retinal diseases, which show a high degree of interocular symmetry.^28^^,^^29^^,^^34^

### Imaging Endpoints

An ideal endpoint for clinical trials should have certain characteristics, including being repeatable and reliable, being identified/acquired in a good proportion of patients, having clinical significance and impact on the quality of life, and to be able to identify a change within the time frame of the trial (1-3 years).^35^ FAF and OCT endpoints are meaningful in progressive diseases because they can indicate relative stability or arrested degeneration in the treated eye. Less than one-quarter of the patients (23.5%) changed the FAF group, and just over one-third of the patients (36.1%) changed OCT grade over a mean follow-up of 5.9 and 5.46 years, respectively. OCT qualitative assessment was able to identify progression in a greater proportion of patients over slightly shorter follow-up, but given the small number of patients, participating in trials and the long follow-up needed are unlikely to provide a useful primary outcome measure. OCT quantitative measurements can be more sensitive to identifying a change, but were smaller in magnitude in *KCNV2*-retinopathy than in other progressive conditions (eg, *RPE65* and *ABCA4* diseases)^36^^,^^37^ and greater than in more stationary conditions, for example, achromatopsia.^38^ Repeatability and reliability of the measurements in the current study was not evaluated; however, based on recent studies in other inherited retinal diseases that evaluated ONL thickness,^39^ DAF area,^28^ and EZW loss,^37^ it is unlikely that these small OCT changes will be sufficiently robust to exceed the repeatability coefficient.

Another noteworthy observation is the relative proportion of patients with quantifiable FAF and OCT features, with a ring of increased signal being identified in less than half of the cohort (44%), DAF being present only in Group 5 FAF (32.1%), and EZW loss only present in Grade 3 and 4 OCT (combined 28.4%). In direct contrast, ONL thickness can be quantified in a large proportion of patients (Grades 1-4, 75%), who importantly are also likely candidates for potential intervention (eg, lack of foveal atrophy). The identification of a reliable and repeatable OCT or FAF measurement that applies to most of the patients with *KCNV2*-retinopathy may therefore be challenging.

### Window for Intervention

Sergouniotis and associates^17^ suggested a window of opportunity in an OCT study (n = 12), during which novel therapeutic intervention, such as gene augmentation therapy, may rescue retinal function, despite the early structural changes observed.^15^ Report No. 1 identified a normal rate of age-associated change on full-field ERGs, consistent with largely stable peripheral retinal dysfunction across 6 decades.^4^ Stockman and associates^40^ psychophysically characterized the disease and suggested an intact phototransduction process. Patients with retinal atrophy (OCT Grade 5) are unlikely to benefit from gene therapy, given the lack of foveal photoreceptors and the relatively stable peripheral retinal function. Patients with OCT Grades 1-4 have preserved ONL and are likely to benefit from intervention, with the subset of patients with preserved EZ (OCT Grades 1 and 2), having even greater potential. Seventy-five percent of our cohort had OCT Grades 1-4, and almost half of the cohort had Grades 1 and 2 (46%), with an age range of 4-51 years (Table 1). As shown on the boxplots in Figure 3, B, most of the patients with Grades 1-4 are below the age of 40 years, but with an overlap between grades and with no patient developing atrophy before the age of 19 years (Table 1). Overall, the window for intervention is likely to be up to approximately 40 years old, with every patient needing to be assessed individually for the presence of retinal atrophy—given the variability and overlap in grades.

### Future Directions

Investigation of retinal sensitivity measurements and their correlation with the structural findings of *KCNV2*-retinopathy may be a broadly applicable endpoint. Report No. 1 investigated the genetic background of the disease but was not able to identify any definite genotype-phenotype correlations;^4^ these would however be valuable both to inform advice to patients on their prognosis and to potentially aid stratification of trial participants, for example, by their rate of progression/greater disease severity. In addition, the presence of intrafamilial variability is a common feature in inherited retinal diseases and has not previously been explored in *KCNV2*-retinopathy.

Moreover, a pilot study of advanced retinal imaging with adaptive optics has previously identified decreased cone density in *KCNV2*-retinopathy.^15^ Further investigation using imaging systems with greater resolution,^41^ more extensive protocols, and longitudinal prospective assessment will be valuable to better characterize how rescuable the photoreceptor mosaic is in *KCNV2*-retinopathy and also may identify novel structural endpoints. Also, prospective standardized acquisition of OCT and FAF imaging at regular intervals in a large cohort may further elucidate disease natural history, including allowing application of other measurements not explored herein (eg, EZ area) and assessment of “presymptomatic” patients.

### Limitations

Several limitations can be identified in the current study, due to its retrospective and multisite nature. Not all the data were available for all patients, and many of the available data were acquired by different machines (eg, OCT, FAF) and/or different protocols (eg, field of view, follow-up time). Axial length affects transverse scaling of FAF and OCT images,^42^ and it was not available for the cohort for the scaling of EZW loss and DAF and ring area measurements. FAF signal for group classification was evaluated to be increased, normal, and decreased qualitatively (eg, no signal was quantified).

## CONCLUSIONS

This study is the first in-depth analysis and long-term longitudinal study of *KCNV2*-retinopathy. *KCNV2*-retinopathy is a slowly progressive disease with early macular changes and shows a high degree of interocular symmetry in most cases. The identification of an OCT or FAF-based endpoint that applies to the majority of patients with *KCNV2*-retinopathy may be challenging. The window for intervention is wide, up to 40 years of age, making it an attractive target for therapies; however, given the variability in the development of macular atrophy, patients will need to be assessed individually. There is a need for a prospective longitudinal natural history study to further investigate disease progression both structurally and with detailed functional assessments.

## Appendix Supplementary materials

Image, application 1

Image, application 2
