# Supplementary material for: KCNV2-Associated Retinopathy: Detailed Retinal Phenotype and Structural Endpoints—KCNV2 Study Group Report 2
Source: Am J Ophthalmol. 2021 Oct;230:1–11. doi: 10.1016/j.ajo.2021.03.004 (PMC8710866; doi:10.1016/j.ajo.2021.03.004)
Supplement: Supplementary file 1 [file mmc1.pdf]

Supplementary Material 1: Methods

Fundus Autofluorescence (FAF) Quantitative Analysis

The observer selected seed points within candidate areas of atrophy by clicking inside the lesion, on the darkest areas. The software considers all the adjacent pixels with a signal intensity equal to and below the signal intensity of the seed point and outlines the region. An observer makes adjustments by changing the threshold of the region-growing algorithm to precisely outline the region. In the presence of multifocal lesions, the sum of all areas of decreased AF (DAF) were calculated. In this study we have used previously established conventions: (i) the minimum lesion size considered as an area of DAF was defined to have a diameter greater than 125 μm and/or a lesion area of 0.012 mm^2; (ii) shadow correction was applied when the FAF images were unevenly or inadequately illuminated; (iii) manual line, circles, contours or free-hand constraints were used as needed to distinguish lesion boundaries and exclude vascular structures; (iv) peripapillary atrophy was excluded from area calculation; (v) in case of confluence of central and peripapillary atrophy, an approximately vertical line constraint had to be set at the narrowest part (“bridge”), with atrophy quantification including only atrophy temporal to the constraint; and (vi) areas of foveal sparing were delineated with free-hand constraints after consulting infrared images and/or OCT obtained at the same visit.

Optical Coherence Tomography (OCT) Retinal Location Identification

The foveal center was identified on the baseline transfoveal OCT image and the corresponding point was marked on the accompanying baseline NIR-R fundus image. The baseline NIR-R overlay is copied and pasted on the final NIR-R fundus image as

per vendor software, after aligning them. The vertical OCT marker position on the final image was then adjusted to correspond to that shown on the final NIR-R image, so the line scan over the exact same location was identified.
