# Supplementary material for: KCNV2-Associated Retinopathy: Detailed Retinal Phenotype and Structural Endpoints—KCNV2 Study Group Report 2
Source: Am J Ophthalmol. 2021 Oct;230:1–11. doi: 10.1016/j.ajo.2021.03.004 (PMC8710866; doi:10.1016/j.ajo.2021.03.004)
Supplement: Supplementary file 2 [file mmc2.pdf]

Supplementary Material 2: Figure

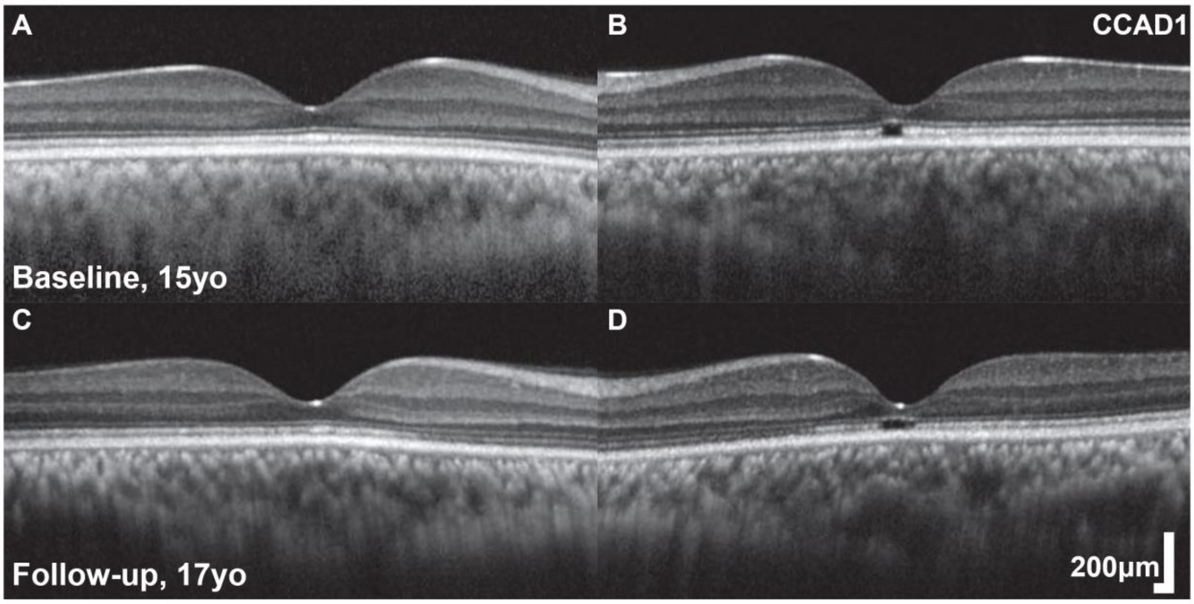

Figure: Disease asymmetry in a case of *KCNV2*-retinopathy.

A 15-year-old child presented with asymmetric disease at baseline on optical coherence tomography (OCT) imaging (A-B) and preserved the interocular difference over 2 years of follow-up (C-D). The right eye (A and C) had a continuous ellipsoid zone (Grade 1), while the left eye (B and D) had a hyporeflective zone (optical gap, Grade 4).

yo; years old
